# Comparison of the short-term effects of lumbar endoscopic and microscopic tubular unilateral laminotomy with bilateral decompression in the treatment of elderly patients with lumbar spinal stenosis

**DOI:** 10.1186/s40001-022-00847-0

**Published:** 2022-10-29

**Authors:** Jianing Zhang, Dingjie Liang, Mengmeng Xu, Kun Yan, Dapeng Zhang, Weiqing Qian

**Affiliations:** 1 NanJing Hospital of Chinese Medicine Affiliated to Nanjing University of Chinese Medicine, Nanjing, 210001 Jiangsu China; 2 Nanjing University of Chinese Medicine, Nanjing, 210001 Jiangsu China; 3 Department of Orthopedic, NanJing Hospital of Chinese Medicine Affiliated to Nanjing University of Chinese Medicine, 157 Da Ming Road, Nanjing, China

**Keywords:** Lumbar spinal stenosis, Elderly, Quadrant channel, Delta large channel endoscopy

## Abstract

**Objective:**

To compare the clinical efficacy of lumbar endoscopic Delta large channel and microscopic tubular Quadrant channel unilateral laminotomy with bilateral decompression in the treatment of elderly patients with lumbar spinal stenosis.

**Methods:**

A total of 40 patients aged above 75 years with lumbar spinal stenosis admitted from June 2019 to August 2021 were reviewed, in which the observation group was treated with the Delta large channel technique and the control group was treated with Quadrant channel open decompression. The general data, duration of illness, operation time, intraoperative bleeding, VAS score preoperatively, 3 days postoperatively, 3 months postoperatively and 6 months postoperatively, and ODI index of the two groups were recorded in the two groups.

**Results:**

The observation group had significantly shorter operation time (59.93 ± 10.46 min vs 77.66 ± 12.44 min, *P* < 0.001) and less intraoperative bleeding (21.06 ± 4.59 mL vs 51.00 ± 10.02 mL, *P* < 0.001) than the control group. There were no significant differences between the two groups in the duration of illness (11.85 ± 5.08 years vs 13.80 ± 7.40 years, *P* = 0.337), VAS score preoperatively (6.05 ± 1.19 vs 6.40 ± 1.47, P = 0.412), 3 days postoperatively (1.90 ± 0.85 vs 2.00 ± 1.08, *P* = 0.746), 3 months postoperatively (1.10 ± 0.31 vs 1.20 ± 0.41, *P* = 0.389) and 6 months postoperatively (1.25 ± 0.44 vs 1.30 ± 0.57, *P* = 0.759), and ODI index preoperatively (0.78 ± 0.07 vs 0.74 ± 0.07, *P* = 0.09), 3 months postoperatively (0.28 ± 0.06 vs 0.30 ± 0.05, *P* = 0.189) and 6 months postoperatively (0.21 ± 0.07 vs 0.22 ± 0.04, *P* = 0.444) (*P* > 0.05). The ODI index 3 days postoperatively in the observation group was significantly lower than that in the control group (0.33 ± 0.06 vs 0.37 ± 0.05, *P* = 0.022).

**Conclusion:**

Both surgical methods had good clinical outcomes for the treatment of lumbar spinal stenosis. However, Delta large channel endoscopy had a clearer vision, less trauma and lower incidence of early postoperative back pain than that of Quadrant channel open decompression.

## Introduction

Lumbar spinal stenosis (LSS) is defined as narrowing of the lumbar central canal, nerve root canal, lateral saphenous fossa, or intervertebral foraminal cavity due to various causes, resulting in compression of the spinal cord, dural sac, and neurovascular vessels, and eventually leading to the corresponding clinical symptoms [1]. It is primarily manifested as intermittent claudication of neurogenic origin, accompanied by lumbar pain and radiological pain, numbness and weakness of the lower limbs. Therefore, it is also called as lumbar spinal stenosis syndrome [2]. Symptoms are aggravated by walking or prolonged standing and relieved by forward bending or sitting [3]. In the past, the main treatment methods for LSS included conservative treatment and posterior open surgical treatment. The conservative treatment refers to the treatment by anti-inflammatory and analgesic drugs [4]. Although this method can relieve the pain of patients, it fails to fundamentally solve the problem. Especially for elderly patients, conservative treatment is often less effective because of the long duration of illness and the thickening of the ligamentum flavum, calcification and bony hyperplasia of the articular eminence are more serious [5]. Open surgery can be performed by fully exposing the posterior lumbar structures for adequate decompression, repositioning, and fusion fixation. However, most elderly patients are unable to tolerate open surgery due to its shortcomings, such as high trauma, high complication rate, and slow postoperative recovery [6].

The emergence of Quadrant channel open decompression has well solved this contradiction, which achieves complete decompression while greatly reducing the surgical trauma. With the continuous advancement of minimally invasive techniques, Delta large channel endoscopy has been created and was used for minimally invasive treatment of LSS in elderly patients. However, few studies [7, 8] have investigated the clinical efficacy of Delta large channel endoscopy and Quadrant channel open decompression in the treatment of LSS in elderly patients. In this study, we retrospectively analyzed the clinical efficacy of Delta large channel endoscopy in a cohort of 40 elderly LSS patients, using the Quadrant channel open decompression as the reference technique as previously reported [9].

## Materials and methods

### General materials

A total of 40 LSS patients aged above 75 years admitted from June 2019 to August 2021 were selected and included in this study.

### Inclusion criteria for cases

(1) Aged above 75 years; (2) neurogenic intermittent claudication as clinical symptom with or without radiculopathy and single segment LSS diagnosed by imaging; (3) unsatisfactory relief of symptoms such as low back pain and intermittent claudication after systematic conservative treatment (more than 3 months); (4) no contraindications to surgery; (5) able to complete 6-month follow-up; (6) patients and their families voluntarily signed the informed consent forms.

### Exclusion criteria

(1) Patients with a history of previous lumbar spine related surgery or fracture; (2) those with spinal tumors and infectious diseases such as tuberculosis; (3) those with serious heart, liver and kidney impairment disease; (4) those with symptomatic multi-segmental lesions of the lumbar spine; (5) those who were unable to cooperate to complete in the 6-month follow-up.

All patients were divided into the observation group and the control group according to the inclusion criteria and different treatment methods, in which the observation group was treated by Delta large channel technique and the control group was treated by Quadrant channel open decompression, with 20 patients in each group.

### Treatment methods

**Fig. 1:**
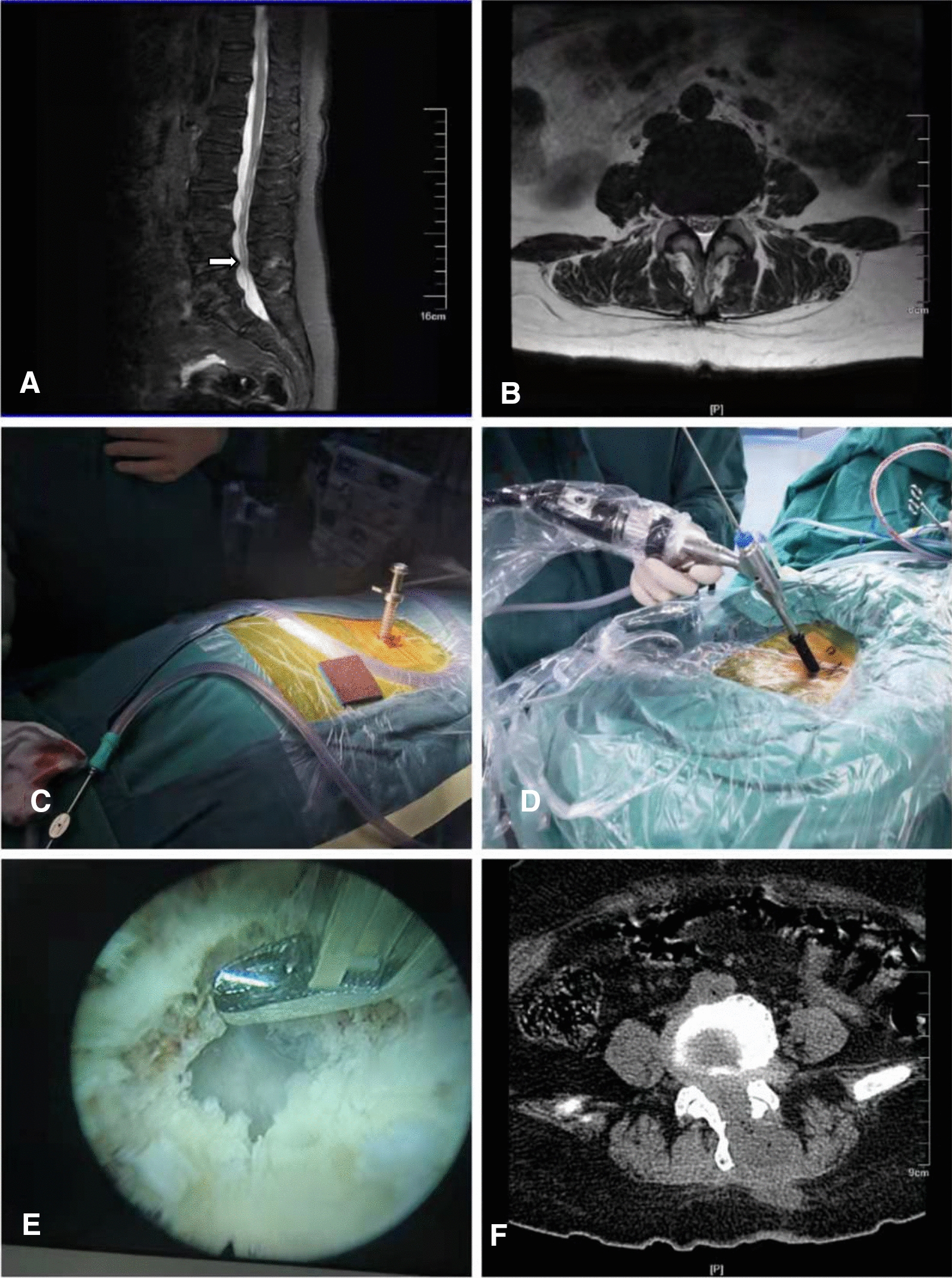
The imaging data of a 76-year-old woman with LSS treated by Delta large channel endoscopy. **A**,** B** Preoperative MRI showed L4/5 spinal canal stenosis. **C**, **D** Patient's position and established operating channel. **E** Exposure of the nerve roots and dural sac of the L4/5 segment. **F** Postoperative cross-sectional CT showed adequate decompression of the L4/5 spinal canal. White arrow indicates level of decompression

Observation group [10]: After successful general anesthesia, the patient was fixed on the operating table in a prone position with the chest and iliac bone partially elevated and the target vertebral space positioned on the body surface. The skin was routinely disinfected and covered with sterile cavity wipes. The positioning needle was placed at about 2 cm of paracentral opening of the spinous process. The C-arm machine determined that the positioning needle was located at the midpoint of the vertebral plate space at the inner edge of the articular eminence. A guide wire was inserted and the skin was incised at the insertion point, approximately 1 cm in diameter. The dilatation tube was placed along the incision step by step, and finally the working sleeve was placed to establish the working tube. The working sleeve should be well positioned with the C-arm machine, and connected with the light source, endoscope and flushing saline. The fluoroscope was placed into the working tube and the appropriate water flow was adjusted. The microscopic radiofrequency tip was used for the processing of the soft tissue on the superior and inferior edges of the lamina and on the surface of the ligamentum flavum. A microscopic power grinder was placed to endoscopically grind the lower edge of the superior lamina and the upper edge of the inferior lamina. A punch forceps was used to bite off part of the lamina and widen the lamina gap, thus revealing the ligamentum flavum. The ligamentum flavum was excised from the center to the lateral side with punch forceps to expose the dural sac and the lateral edge of the nerve root, and if necessary, part of the articular eminence was excised to fully expose and release the nerve root for lateral saphenous fossa decompression; followed by radiofrequency hemostasis. The herniated intervertebral disc of the diseased segment was observed, the nucleus pulposus with grasping forceps was slowly removed, and the intervertebral space was cleared. After sufficient decompression, radiofrequency ablation and fibrous annuloplasty were performed. Finally, the working channel was withdrawn, the surgical incision was sutured, and the adjuvant patch was applied, as shown in Fig. 1.

**Fig. 2:**
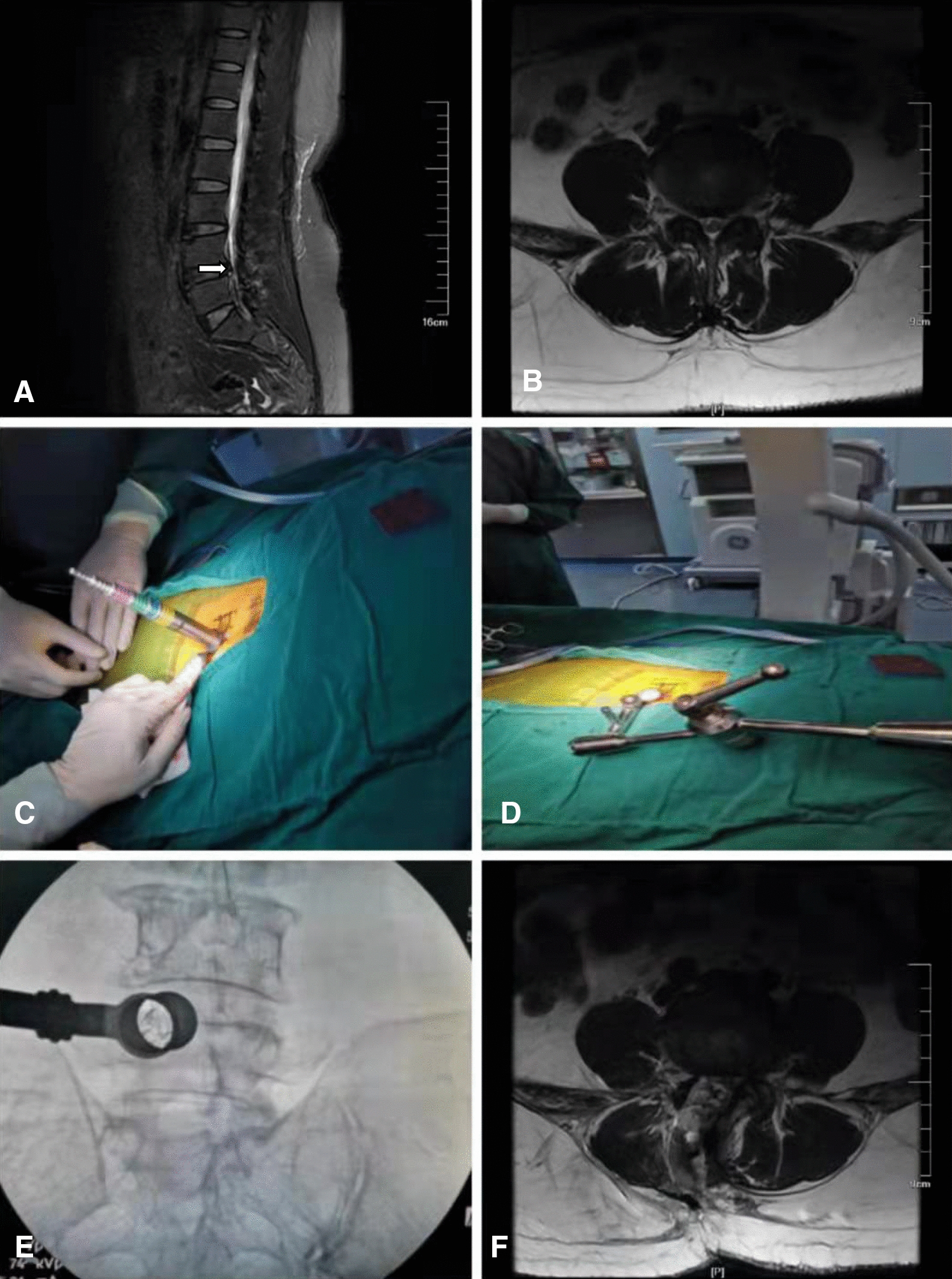
The imaging data of a 78-year-old man with LSS treated with Quadrant channel open decompression. **G**, **H** Preoperative MRI showed L4/5 spinal canal stenosis. **I**, **J** The patient's posture and operating channel and fixing devices. **K** Intraoperative positive and lateral X-ray showed the situation of the tube placement. **L** Postoperative cross-sectional CT showed adequate decompression of the L4/5 spinal canal. White arrow indicates level of decompression

Control group [11]: After successful general anesthesia, the patient was fixed on the operating table in a prone position, with pillow cushioned on the chest and hips. The surgical site was further identified by C-arm fluoroscopy, routinely disinfected with 2.5% iodophor, and routinely laid with surgical towels. After positioning under fluoroscopy, the guide needle was inserted, and the corresponding puncture needle was removed. The skin, subcutaneous tissue and lumbar fascia were incised sequentially at approximately 3 cm away from the spinous process of the affected lesion, and the paravertebral muscles were separated with a Quadrant instrument and placed into the outer edge of the patient's lamina as well as the articular facet joint to establish a channel. On the affected side of the lesioned segment, the lower edge of the upper lamina and the upper edge of the lower lamina were exposed. Subsequently, the patient’s lower vertebral joint and the superior half of the upper articular process of the lower vertebral body were removed with an electric grinder to "open the window". Obvious hypertrophy of the ligamentum flavum, partial adhesion of the dura mater, and stenosis of the central spinal canal were found. The hypertrophic ligamentum flavum was removed and the central spinal canal was decompressed. The herniated disc was seen in the lesion segment, which compressed the nerve root on the affected side. The herniated nucleus pulposus was removed using instruments such as nerve probes, nerve hooks, grasping forceps, and biting forceps, and the fibrous ring breach was scarified with an electric knife. The nerve root on the affected side was adequately decompressed on re-exploration. After the procedure was completed, the working channel was removed, the incision was flushed with saline, and the wound was sutured after a drainage tube was left in place, as shown in Fig. 2.

### Efficacy and scoring standards

The gender, age, duration of illness, operation time, and intraoperative bleeding of the two groups were recorded in the two groups. The VAS score and ODI index of the two groups were recorded.

### Statistical method

The collected data were analyzed by SPSS 25.0 and presented by x ± s or case(percentage), as appropriate. The data were compared by independent-samples t-test or Chi-square test or Fisher’s exact test, as appropriate. A two-sided P value less than 0.05 denoted statistical significance.

## Results

### General information

There were 5 male patients and 15 female patients in both groups. The mean age was 81.10 ± 3.44 years in the observation group and 82.65 ± 5.12 years in the control group. There were no significant differences in terms of gender, age, presence of radiculopathy, co-morbidities of hypertension and diabetes, and duration of illness between the two groups (*P* > 0.05), as shown in Table 1.

**Table 1:** Comparison of demographics and clinical characteristics between the 2 groups

Variables	Observation group (*n* = 20)	Control group (*n* = 20)	*P* value
Age (years)	81.10 ± 3.44	82.65 ± 5.12	0.269
Gender (n, %)			
Male	5 (20.00)	5 (20.00)	> 0.999
Female	15 (80.00)	15 (80.00)	
Disease duration (years)	11.85 ± 5.08	13.80 ± 7.40	0.337
Radiculopathy (*n*, %)			
Yes	13 (65.00)	11 (55.00)	0.519
No	7 (35.00)	9 (45.00)	
Obesity (*n*, %)			
Yes	8 (40.00)	6 (30.00)	0.507
No	12 (60.00)	14 (70.00)	
Hypertension			
Yes	7 (35.00)	9 (45.00)	0.519
No	13 (65.00)	11 (55.00)	
Diabetes			
Yes	3 (15.00)	6 (30.00)	0.256
No	17 (85.00)	14 (70.00)	
Pre-operative VAS score	6.05 ± 1.19	6.40 ± 1.47	0.412
Pre-operative ODI	0.78 ± 0.07	0.74 ± 0.07	0.090

*ODI* Oswestry Disability Index

### General results

The observation group had significantly shorter operation time (59.93 ± 10.46 min vs 77.66 ± 12.44 min, *P* < 0.001) and less intraoperative bleeding (21.06 ± 4.59 mL vs 51.00 ± 10.02 mL, *P* < 0.001) than the control group, as shown in Table 2. No significant differences were noted with regard to the number of intraoperative X-ray exposures, level of surgery and postoperative complications (nerve root injury, dural sac injury, hematoma and incision infection).

**Table 2:** Comparison of operative and post-operative data between the 2 groups

Variables	Observation group (*n* = 20)	Control group (*n* = 20)	*P* value
Operation time (min)	59.93 ± 10.46	77.66 ± 12.44	< 0.001
Volume of blood loss (ml)	21.06 ± 4.59	51.00 ± 10.02	< 0.001
No. of X-ray exposures	24.54 ± 4.50	22.03 ± 4.12	0.073
Level of surgery (*n*, %)			0.327
L4/5	9 (45.00)	6 (30.00)	
L5/S1	11 (55.00)	14 (70.00)	
Post-operative complications (*n*, %)	2 (10.00)	4 (20.00)	0.661
Post-operative day 3 VAS	1.90 ± 0.85	2.00 ± 1.08	0.746
Post-operative months 3 VAS	1.10 ± 0.31	1.20 ± 0.41	0.389
Post-operative months 6 VAS	1.25 ± 0.44	1.30 ± 0.57	0.759
Post-operative day 3 ODI	0.33 ± 0.06	0.37 ± 0.05	0.022
Post-operative months 3 ODI	0.28 ± 0.06	0.30 ± 0.05	0.189
Post-operative months 6 ODI	0.21 ± 0.07	0.22 ± 0.04	0.444

### Comparison of VAS Score

As shown in Table 1, there was no significant difference between the two groups in the VAS score preoperatively (*P* = 0.412), 3 days postoperatively (*P* = 0.749), 3 months postoperatively (*P* = 0.389) and 6 months postoperatively (*P* = 0.759). The postoperative VAS score in each group was significantly lower than that before the operation (*P* < 0.05, Table 2).

### Comparison of ODI Index

As presented in Tables 1 and 2, there was no significant difference between the two groups in the ODI index preoperatively (*P* = 0.09), 3 months postoperatively (*P* = 0.189) and 6 months postoperatively (*P* = 0.444). The ODI index 3 days postoperatively in the observation group was significantly lower than that in the control group (0.33 ± 0.06 vs 0.37 ± 0.05, *P* = 0.022).

## Discussion

LSS characterized by intermittent claudication is a common degenerative disease of the lumbar spine. With the progression of disease, the patient's walking distance is gradually decreased, which will seriously affect the quality of life of the patient eventually [12]. It is the main cause of low back pain and even bilateral lower limb paralysis in the elderly [13]. The main causes of LSS in elderly patients are the dysfunction of blood circulation caused by compression and the effects of inflammatory factors. Due to the morphological transformation of lumbar bone and its surrounding soft tissue, the increase of intraspinal pressure results in the compression of spinal cord or nerve root, which in turn leads to the aggravation of the disease [14]. Conservative treatment and surgical treatment are used in the clinical treatment of LSS. Conservative treatment, such as oral analgesics, transforaminal epidural block and selective nerve root block can potentially allow for the spontaneous total resolution of symptoms in young patients [15]. The surgical treatment includes traditional posterior open surgical and minimally invasive channel therapy. The safety of surgery is particularly critical in elderly patients as these people are often complicated with a variety of medical diseases, and their physiological function is significantly deteriorated, making it poorly tolerated, with high surgical risks, high complication rates, and a greater chance of dural injury. Therefore, it is necessary for the operator to make a reasonable choice of surgical approach according to the surgical indications, so that elderly patients can also receive surgical treatment. Due to a large exposed area of traditional open surgery, the patients are prone to great bleeding, increasing the risk of infection, and destroying the structure of facet joints, which harms the stability of the patient's spine and greatly increase the chance of postoperative low back pain. As a result, it is difficult to be tolerated by elderly patients and was not conducive to their recovery. Therefore, orthopedic doctors urgently need to find a new surgical method to make up for the shortcomings of open surgery.

With the development of medical technology, minimally invasive channel surgery has gradually developed. Minimally invasive microscopic or endoscopic decompression surgery can fully expand the spinal canal which can avoid the shortcomings of traditional posterior open surgery such as large openings and incomplete decompression [16]. At present, there is still a great debate about the surgical treatment of LSS in the elderly. Compared with traditional posterior open surgery, Quadrant channel open decompression has the advantages of shorter operation time, less intraoperative bleeding, less postoperative lumbar spine slippage and degeneration, and lower postoperative infection rate [17]. Besides, Quadrant channel open decompression creates a working channel through the continuous expansion using the working trocar needle, which can eliminate the need for extensive dissection of the paravertebral muscles to reveal skeletal landmarks [18], making it one of the effective treatment methods for LSS. Due to the large pressure on the surrounding soft tissues during the placement of the step-by-step expansion channel, however it is easy to cause local skin and subcutaneous tissue necrosis at the operation site if the operation is performed for a long time, which will affect the healing of the incision [19]. In addition, when the channel is installed, muscle and joint capsule tissue will be left to varying degrees at the bottom of the channel due to the influence of the articular process. To obtain a better exposure, therefore, the soft tissue at the bottom of the channel needs to be removed inevitably during the surgery, which may lead to the injury of the muscle attachment points and joint capsule of the surgical segment, and easily result in postoperative chronic low back pain. At the same time, due to the limitation of the channel, the surgical field of vision is small, coupled with intraoperative blood seepage, and the clarity and comfort of the surgical field of vision is poor, so it is often necessary to use a special surgical microscope. Besides, the requirements for accuracy in positioning are more stringent, and the operation time is relatively long, which is easy to cause muscle fatigue of the operator [20], thus increasing the technical difficulty of the operation for the surgeon. Some researchers have suggested that a “precise” fenestration operation should be performed under the intervertebral foraminal microscope for decompression, which aims to effectively alleviate the disease with minimal trauma [21], so percutaneous transforaminal endoscopic technique has emerged [22]. However, the intervertebral foraminal microscope cannot fully reduce the pressure for the severe LSS. The emergence of Delta large channel endoscopy technology responds to the requirements of development. The Delta endoscope is further optimized and improved on the basis of the traditional foraminal endoscope, and the scope of decompression under the endoscope is further expanded by expanding the working channel [23]. It also has a large diameter, simple operation, and has a field of view similar to that of open surgery [24], as well as equipped with a larger grinding drill that is large enough to easily remove excess bone and enlarge synapses [25], which avoids the shortcomings of the limited field of view in Quadrant channel open decompression. Moreover, with a large operation space and less damage to the muscle attachment point and joint capsule of the surgical segment, the risk of postoperative low back pain is greatly reduced. The report of Wu et al. showed that the Delta large channel endoscopy technology has a significant effect on the treatment of LSS [26], which can not only release the compression of the nerve by the dural sac and the bone around the nerve root, but also release the compression of soft tissues such as the intervertebral disc, posterior longitudinal ligament and ligamentum flavum, and without obvious damage to the lumbar spine [27], thereby effectively alleviating the the patient's low back and bilateral lower extremity radiological pain and improving the body function of the patient. Moreover, it can shorten the operation time and reduce the amount of intraoperative bleeding, and uses water as the operating medium, which can make the field of vision clearer [28]. During the operation, continuous normal saline irrigation can flush out various inflammatory mediators around the diseased intervertebral disc and the by-products left by electrocoagulation, which can relieve postoperative pain [29].

In this study, all the 40 patients successfully completed the surgery. 19 out of the 20 patients in the observation group had no postoperative complications, and 1 case of surgical site infection occurred, which was cured after symptomatic treatment with anti-inflammatory and standard drug change. In the control group, 15 out of 20 patients had no postoperative complications. 1 case of cerebrospinal fluid leakage occurred, which was cured after bed rest and conservative treatment with antibiotics; 3 cases of low back pain were cured after treatment with anti-inflammatory and analgesic drugs; 1 case of limited necrosis at the skin edge of the surgical incision was cured after drug change. Ultrasonic bone knife was used during the Quadrant channel open decompression operation, whose vibration would generate heat. Due to the small incision, it was inevitable to contact the skin during the operation. Therefore, the heat generated by the ultrasonic bone knife might burn the skin in contact, which resulted in skin infection and necrosis.

In summary, Quadrant channel open decompression can shorten the operation time, reduce intraoperative bleeding, and reduce surgical trauma [30]. It has a short learning curve, and has a strong three-dimensional sense and overall sense during the operation. However, the surgical field of view is small, special operating microscopes are required, and the probability of postoperative chronic low back pain is high.

The emergence of Delta large channel endoscopy has made up for the shortcomings of the Quadrant channel open decompression, with less damage to the patient and a better curative effect and obvious advantages in relieving back and leg pain and improving patients' daily life status. It greatly shortens the bed rest and hospitalization time, and has a low incidence of complications, which greatly ensures the quality of life of patients, and is worthy of clinical promotion. Although the Delta large channel endoscopy has a long learning curve and certain limitations [31]; for example, when the interlaminar approach is placed in the working channel, the dura mater is sometimes compressed, and the nerve root is stretched and injured and excessive saline flushed too fast intraoperation can lead to increased epidural pressure. However, as a modification and optimization of the total spine endoscopy, it has more potential for development in terms of trauma, visual field and work efficiency. There were still some shortcomings in this study. For example the sample size was small and the clinical observation time was short. There might be some deviations in the report on the treatment effect, which needed to be further studied at a later stage. In addition, the treatment should be comprehensively considered according to the patient's body condition and economic conditions.

## Data Availability

All data generated or analyzed during this study are included in this published article.
